# Editorial: Addressing contemporary public health challenges in Ghana for improved outcomes: getting to SDG 3

**DOI:** 10.3389/fpubh.2024.1461659

**Published:** 2024-09-04

**Authors:** Delanyo Dovlo, Evelyn K. Ansah, Kwasi Torpey, Irene A. Agyepong

**Affiliations:** ^1^Ghana College of Physicians and Surgeons, Faculty of Public Health, Accra, Ghana; ^2^Institute of Health Research, University of Health and Allied Sciences, Ho, Ghana; ^3^Department of Population, Family and Reproductive Health, University of Ghana, School of Public Health, Accra, Ghana

**Keywords:** contemporary public health challenges, Ghana, improved outcomes, public health, core functions, low and middle income countries

## Introduction

As the world moves forward in its efforts toward the Sustainable Development Goals (SDG) targets in 2030, decision making and implementation for public health programs to promote, restore, maintain, and improve health outcomes needs to be informed by research evidence generated at local as well as international levels. The need is imperative as the low- and middle-income countries (LIMC) disease burdens evolve from the traditional recognized patterns of mainly communicable diseases and maternal and child health problems to an increasingly sophisticated triple burden incorporating non-communicable diseases (1–4). This supplement's topic therefore aimed to attract a collection of articles mainly but not exclusively related to learning from Ghana, an LMIC in Sub-Saharan Africa's, research experience; to draw lessons for Ghana as well as the wider global community and especially for similarly placed countries in terms of contemporary health challenges and dilemmas as to how to best address them. The focus on an LMIC also had the dual aim of encouraging and promoting LMIC led research and innovation as part of strengthening LMIC capacity and leadership for research to respond to contemporary health challenges. Though the call and focus of this special supplement was on Ghana, two papers are included in the supplement from research in China and Taiwan in East Asia, given the relevance of the themes to contemporary public health in Ghana.

## Conceptual/theoretical framework

The conceptual/theoretical framework within which the papers in this supplement are categorized draws on the concepts of health systems and the core functions of public health. Public health deals with the concern of societies to ensure the conditions in which people can be healthy. The UN's Sustainable Development Goal 3, “ensure healthy lives and promote wellbeing for all at all ages” depends on the effective execution of the core functions of public health for its success. Drawing on the 1988 IOM report (5), we used the concept of assessment (gathering the evidence about the state of health of the population, how and why and the conditions in which populations can stay healthy), assurance (putting in place the service, programs, and interventions to promote, restore and maintain health), and policy development (making of decisions that become authoritative for communities and societies to ensure the promotion, restoration, and maintenance of health) as the three core functions of public health; to categorize the papers in this supplement.

**Figure 1:**
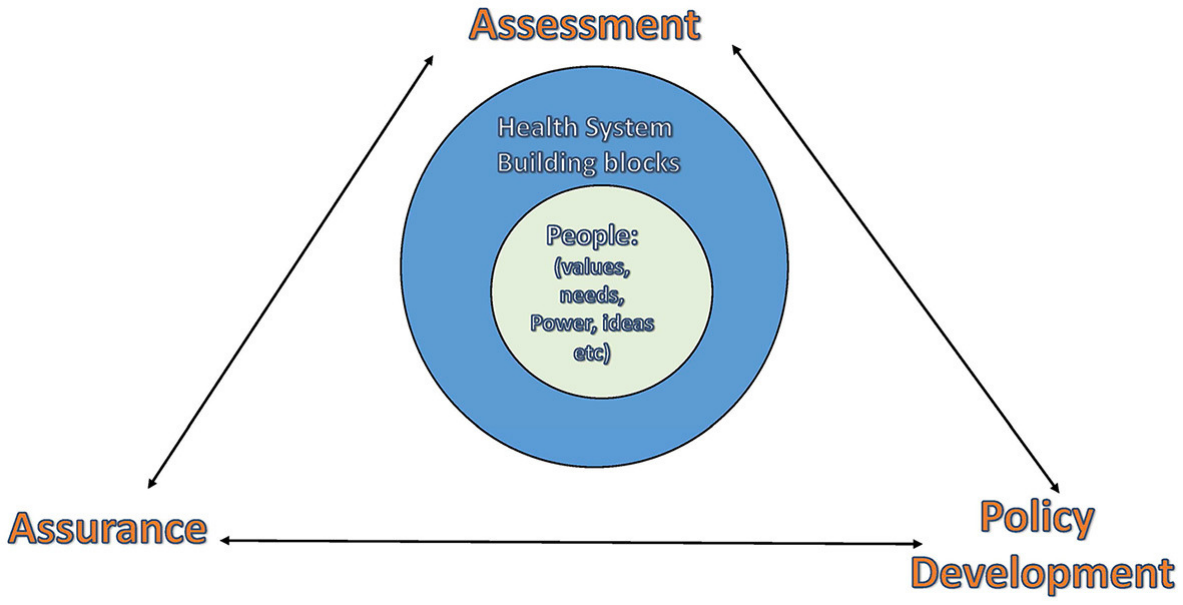
Framework for organizing the papers in the supplement.

We additionally drew on the WHO definition of the health system as referring to all institutions, actors, processes, and actions whose primary intent is to promote, restore and maintain health and contemporary constructs of the health system that go beyond the traditional WHO building blocks and take a social constructivist perspective that recognizes that health systems are social and complex adaptive systems. How the building blocks are structured and function and how the core functions of public health are executed; and the resulting outcomes are influenced by people, their values, interests, ideologies, power, and use of power to influence processes (Figure 1).

Within each category of the categories related to the core functions of public health, we explore whether there is an underlying focus related to the increasingly triple burden faced by LMIC of maternal and child health, communicable, and non-communicable disease.

### Assessment

Of the five papers in the assessment category, all the three papers from West Africa are related to maternal and child health. Salifu et al. (6) evaluate the change in two survey periods of anemia and predictors and contributors to observed trends in Ghana, Sierra Leone, Mali, and Benin. Afagbedzi, Alhassan, Alangea et al. assess maternal factors and child health conditions associated with preterm births. Owusu et al. conduct a district level analysis of the relationship between socio-demographic factors and the COVID-19 pandemic in two regions of Ghana. one paper and another explore maternal factors and child health conditions at birth that are associated with preterm deaths in a tertiary health facility in Ghana. He et al. present findings from an exploration of factors influencing works stress of medical workers in clinical laboratories in China during the COVID-19 pandemic. Their findings confirm the high levels of stress that health workers in the front line of management of a pandemic face and the need to pay attention to reducing the stress on frontline health workers in a health security crisis. Guo et al. present the development of an estimation method for distance cost to access medical services and the policy and patient privacy implications in Taiwan.

### Policy development

The two papers in the supplement are related to the policy development function of public health. Both papers explore the issues in relation to maternal and child health. Agblevor et al. present the results of an exploratory and explanatory study of gaps in implementation of the Ghana Adolescent Health Service Policy and Strategy (2016–2020) and how and why context influenced the observed gaps. Ayim et al. explored local government policy and resource disbursements to support health in the context of decentralization in two districts in the Volta Region of Ghana.

### Assurance

The largest number (7) of the papers are related to the assurance function of public health, and involve evaluation of services, programs and interventions in place to promote, restore and maintain health. Karamagi et al. explore lessons from Ghana on how to assure that districts are functional for UHC attainment. Koduah examines factors that enabled the prioritization and implementation of selected pharmaceutical reform items and how these contributed to improving equitable access to medicines and universal health coverage in Ghana. Elsey et al. in a historical and systematic review of the Community-based Health Planning and Services [CHPS] program in rural and urban Ghana, explore what works for whom and why, observing that despite renewed emphasis on strengthening primary health care globally, it remains under-resourced across sub-Saharan Africa. Awumee and Dery study continuity of care among diabetic patients in Accra, Ghana and it is one of the few papers that looks at a non-communicable disease issue. The use of Long-Lasting Insecticide Nets (LLINs) has been recognized and prioritized as a major intervention for malaria prevention in Ghana. Afagbedzi, Alhassan, Kenu et al. explore the factors influencing the universal coverage and utilization of LLINs in Ghana drawing on data from a cross-sectional survey. The disease focus is thus communicable diseases. Aberese-Ako et al. explore the role of community engagement in COVID-19 management in two Ghanaian municipalities.

Taken together, the collection of articles in this special supplement, bring out the importance of focusing on the increasingly multi-dimensional nature of evolving disease burdens and therefore policy and program priorities in LMIC like Ghana. Without neglecting the traditional priorities of maternal and child health and communicable disease such as malaria in research, policy and program agendas; disease of epidemic potential as well as non-communicable diseases increasingly need more attention.
